# Things and Conditions Are Ever Changing

**Published:** 1897-08

**Authors:** 


### THINGS AND CONDITIONS ARE EVER CHANGING.

The Lancashire Veterinary Medical Association of England recently passed resolutions that "docking" was a necessary operation.

The London *Live Stock Journal*, in one of its July issues, takes cognizance of the dangers arising from the appointment of a non-graduate State Veterinarian in Illinois. How about Michigan?

"Veterinary Consulting Rooms" is the significant heading of an Eastern veterinarian's office stationery.

**Philadelphians can reach Nashville in 36 hours. New Yorkers in 38 hours.**

Six veterinarians attended the great race-horse, "Domino," in his fatal illness from meningitis, at Lexington, Ky.

The Tennessee papers already announce our coming by publicity given to the names of those who expect to read papers, as well as the subjects which they will treat of.

**There will be no section of our country without representation at Nashville.**

The determination to return to the breeding of Morgan horses by the Illinois College of Agriculture will be watched with much interest. Certain families of this well-known breed were among the best all-round horses ever owned in America.

A western city of Pennsylvania is again being placarded by one of her veterinarians with posters 14 x 20 as a hoped-for method of increasing business.

Lincoln has again started the horse-abattoir, which doubtless means the destruction of many cayuses.

